# Mixed nitrogen forms enhance growth and photosynthetic nitrogen use efficiency by optimizing nitrogen metabolism and leaf N allocation in Gisela 6 cherry rootstock

**DOI:** 10.3389/fpls.2025.1696713

**Published:** 2025-11-25

**Authors:** Xinxiang Xu, Yan Tang, Liu He, Yanxia Sun, Daliang Liu, Yuxia Wang, Fangdong Li, Lingling Zhao, Laiqing Song, Fen Wang, Yanju Li, Xu Zhang

**Affiliations:** 1 Yantai Key Laboratory of Apple Germplasm Innovation and Quality Control, Yantai Academy of Agricultural Sciences, Yan’tai, Shandong, China; 2 Xuzhou Nature Environmental Protection Secondary Specialized School, Xuzhou, Jiangsu, China; 3 Key Laboratory of Biochemistry and Molecular Biology in University of Shandong Province, School of Advanced Agricultural Sciences, Weifang University, Weifang, Shandong, China

**Keywords:** nitrate, ammonium, nitrogen metabolism, sweet cherry rootstock, photosynthetic nitrogen use efficiency

## Abstract

Nitrogen (N) is the most important nutrient for plant growth and development. However, the mechanisms by which the form and supply of N regulate the growth and N utilization of cherry rootstock are unclear at present. We investigated the effects of different N supply levels and N forms on the growth, N uptake, assimilation and distribution, and photosynthetic N use efficiency (PNUE) of Gisela 6 cherry rootstock seedlings. The results showed that a high N level and a single supply of either nitrate N or ammonium N hindered N uptake and assimilation, increased photosynthetic limitation, reduced PNUE and ^15^N use efficiency, and inhibited cherry rootstock growth. Further experiments showed that a mixed supply of nitrate N and ammonium N maintained high transcription levels of nitrate and ammonium transporters as well as N metabolism enzyme activities, thereby increasing the net inflow rates of NO_3_^−^ and NH_4_^+^ into roots and the soluble protein content of leaves. In addition, a mixed N supply reduced oxidative damage to leaves by maintaining an appropriate nitrate/ammonium ratio, increased the proportion of leaf N allocated to photosynthetic N, decreased leaf cell wall thickness, and enhanced stomatal conductance, mesophyll conductance, and the maximum carboxylation efficiency. This resulted in reduced leaf photosynthetic limitation, increased leaf net photosynthetic rate and PNUE, and ultimately enhanced the growth of Gisela 6 cherry rootstock seedlings. Our results provide a basis for optimizing N management strategies in cherry cultivation.

## 1 Introduction

Nitrogen (N) is an essential nutrient for plant growth and a fundamental component of metabolites such as nucleic acids, amino acids, and proteins. N promotes root and leaf development, thereby optimizing photosynthesis and improving fruit yield and quality (Krapp, 2015; Qi et al., 2023). N exists in various forms in the soil, with nitrate and ammonium being the primary components of inorganic N and the two main forms absorbed by plants. Different forms of N have distinct effects on plant growth and metabolism (Ding et al., 2021; Wang et al., 2021b). Previous studies have shown that plants may vary in their preference for N forms. When both forms of N are present simultaneously, plants often prefer one over the other. For example, apples and wheat tend to prefer NO_3_^−^ (Li et al., 2013; Zhong et al., 2014), while rice prefers NH_4_^+^ (Cao et al., 2018). Supplying NH_4_^+^ to nitrate-preferring plants can lead to ammonium toxicity, whereas the ability of the roots of ammonium-preferring plants to absorb NO_3_^−^ may be degraded (Kronzucker et al., 1997; Britto and Kronzucker, 2002). Plants’ preferences for N forms are not fixed but reflect a combination of a wide range of dynamic environmental conditions and physiological factors. Changes in external environmental conditions can also affect plants’ selective absorption of NH_4_^+^ and NO_3_^−^ (Warren, 2009). Huang et al. (2018) found that under drought conditions, the expression level of ammonium N transporter proteins in the roots of *Malus hupehensis* Rehd significantly increased, resulting in a relative increase in NH_4_^+^ absorption. Liu et al. (2017) found that tomato seedlings increased the demand for NH_4_^+^ under low-temperature conditions. Other studies have shown that compared with NO_3_^−^, NH_4_^+^ plays a more crucial role in plants’ resistance to salt stress, alleviating the damage to plants (Hessini et al., 2019). Under alkaline soil conditions, plants prefer to absorb NO_3_^−^, a pattern that may be related to different transport mechanisms of NH_4_^+^ and NO_3_^−^ (Hawkins and Robbins, 2010). Currently, there is limited research on the effects of N levels and N forms on the growth, N uptake, and distribution of cherry rootstock, and the cherry rootstock’s preference for N forms remains to be studied.

Photosynthetic N use efficiency (PNUE) refers to the ratio between the photosynthetic rate of leaves and the N content per unit of leaf area. PNUE is generally positively correlated with plant N use efficiency because N uptake, assimilation, and transport all require the energy and carbon skeletons provided by photosynthesis (Yin et al., 2019). Recent studies have found a close relationship between PNUE and N distribution within plants, particularly the distribution of N in leaves (Kang et al., 2024). The leaf N distribution refers to the proportion of N allocated among various cellular structures and free compounds within leaves. Studies have shown that optimizing the leaf N distribution can enhance photosynthetic capacity by up to 60% without increasing N input (Xu et al., 2012; Ali et al., 2016). Changes in the leaf N distribution are determined not only by intrinsic characteristics but also by environmental factors. Under drought conditions, plants may increase the proportion of N allocated to structural components such as cell walls, thereby enhancing cell wall thickness, reducing evaporation, and improving drought resistance (Zhong et al., 2019; Liu et al., 2022). Nutrient supply also significantly affects the leaf N distribution. An excessive N supply leads to a greater allocation to non-photosynthetic N, reducing the proportion allocated to photosynthetic N (Hou et al., 2019). Wei et al. (2022) showed that compared with ammonium addition, nitrate treatment increased the proportion of N allocated to the photosynthetic system within the leaves of *Leymus chinensis* while reducing the amount allocated to cell walls. The leaf N distribution affects leaf growth, the chlorophyll content, and the intensity of photosynthesis. Therefore, understanding changes in leaf N distribution is significant for improving N efficiency and reducing N fertilizer input.

Due to its yield-increasing effects and relatively low price, farmers often overuse N fertilizer in production. China’s annual input of N fertilizer for agriculture during the past decade has been approximately 29.56 million tons, accounting for about 30% of the global agricultural N fertilizer usage, making China the country with the largest application of N fertilizer worldwide (FAO, 2020). The excessive application of N fertilizer not only reduces utilization efficiency and fruit quality but can also cause serious environmental problems, including soil acidification, eutrophication of water bodies, and air pollution, thus hindering the sustainable development of agriculture (Liu et al., 2013, 2021; Wang et al., 2021a). High-density dwarf cultivation is the mainstream trend in modern fruit tree cultivation. As an important dwarfing rootstock, Gisela 6 cherry rootstock is widely adopted due to its advantages such as early fruiting, high yield, and broad adaptability. However, there is limited research on the effects of N levels and forms in cherry rootstock. We hypothesized that an optimal supply of mixed N forms would improve PNUE by enhancing N absorption and assimilation, as well as increasing the proportion of leaf N allocated to photosynthesis. Our findings provide new insights for the rational application of N fertilizer in cherry production.

## 2 Materials and methods

### 2.1 Growth conditions and treatments

The experiment was carried out in a growth chamber at the Yantai Academy of Agricultural Sciences between March and May 2024. The test subjects were Gisela 6 sweet cherry rootstock. Uniformly grown rootstocks (approximately 8 cm in height) were selected and planted in plastic pots filled with vermiculite, with one plant per pot. There were 40 replicates for each treatment, with one rootstock per replicate, totaling 240 pots. After transplanting, the seedlings underwent a one-week recovery period during which they were irrigated with a half-strength Hoagland’s nutrient solution. The formal trial began on March 20th.

In the pre-experiment, four N supply levels (5, 10, 15, and 20 mM) were tested, and the rootstocks grew best at 10 mM (**Supplementary Table S1**). Thus, we selected two N supply levels (10 and 20 mM) and three N forms (nitrate, ammonium, and a mixture of nitrate and ammonium) for treatment in the formal experiment. For the nitrate treatment, Ca(NO_3_)_2_ was used as the sole N source; for the ammonium treatment, (NH_4_)_2_SO_4_ was used as the sole N source; and for the mixed N treatment, both (NH_4_)_2_SO_4_ and Ca(NO_3_)_2_ were used, at an NH_4_^+^:NO_3_^−^ ratio of 1:1. There were a total of six treatments: medium N with nitrate (NN10), medium N with ammonium (AA10), medium N with mixed N (N5A5), high N with nitrate (NN20), high N with ammonium (AA20), and high N with mixed N (N10A10). A summary of six N treatments is provided in **Supplementary Table S2**. CaCl_2_ was used for calcium (Ca) supplementation to ensure that the Ca levels were the same across all treatments. The concentrations of other nutrients were similar to those in the Hoagland’s nutrient solution, with these concentrations remaining consistent across all treatment groups.

The nutrient solution treatments were washed every three days with deionized water before each application to remove any residue from the previous feeding. For each treatment, five seedlings were selected for ^15^N isotope labeling. During each application of the nutrient solution, 0.02 g of Ca(^15^NO_3_)_2_ was added to each pot, and this process was repeated 10 times for a total of 0.2 g of Ca(^15^NO_3_)_2_ used for ^15^N labeling. After 45 days of treatment, seedlings were taken to determine various indices.

### 2.2 Analysis of NO_3_^−^and NH_4_^+^ flow rates in roots

The NO_3_^−^ and NH_4_^+^ net ion fluxes were analyzed with a scanning non-invasive micro-test technique system (NMT 100 Series, USA). Briefly, the roots were washed with ultrapure water and placed in plastic dishes. The measuring solution was added until the roots were submerged. The composition of the measuring solution is listed in **Supplementary Table S3**. Each root was tested continuously for 10 min, with six replicates per treatment. The ionic flux data were calculated using MageFlux (imFluxes V2.0). NO_3_^−^ and NH_4_^+^ flux data with positive values represented efflux, and negative values represented influx.

### 2.3 Analysis of growth parameters, ^15^N isotope and N content

After 45 days of treatment, the rootstocks were destructively sampled and categorized into leaves, stems, and roots. The analysis of root morphology was conducted using WinRhizo software (version 2012b, Regent Instruments Canada). The samples (leaves, roots, and stems) were dried at 80 °C to a constant weight, and each part was then weighed using an electronic balance with a precision of 1/1000. Then they were processed through a 0.25 mm mesh screen for grinding and filtering. The abundances of ^15^N isotopes were measured using a MAT-251 stable isotope ratio mass spectrometer. The calculations were performed according to the methodology outlined by Xu et al. (2020). The dried samples were powdered and digested with a mixture of H_2_SO_4_ and H_2_O_2_. A Kjeldahl apparatus (model JK9870) was used to measure the N content.

The calculation formula of the ^15^N distribution rate and ^15^N use efficiency were as follows:


N 15 distribution rate (%) =N 15 absorbed by each organ from fertilizer (mg)total N 15 absorbed by plant from fertilizer (mg)×100%

N 15 use efficiency(%) =total N 15 absorbed by plant from fertilizer (mg)N 15 in fertilizer×100%

### 2.4 Photosynthetic parameters and photosynthetic N allocation

After 40 days of treatment, gas exchange parameters and *P*_n_−C_i_ curves of the fourth leaf on the main stem were recorded between 9:00 AM and 11:30 AM using a LI-6400 portable photosynthesis system (LI-COR Inc., USA). The *V*_max_ and *J*_max_ were computed based on the method described by Long and Bernacchi (2003). Mesophyll conductance (*g*_m_) was calculated following the procedure outlined by Harley et al. (1992). The photosynthetic limitation was calculated according to the method described by Lu et al. (2019).

Based on the system described by Niinemets and Tenhunen (1997), photosynthetic N (*N*_psn_) in leaves was categorized into three main components: N allocated to proteins involved in carboxylation during the *N*_cb_, *N*_lc_, and *N*_et_. The calculations of *N*_psn_ and PNUE were performed following Xu et al. (2024).

### 2.5 Leaf structure analysis

Scanning electron microscopy (SEM) for the leaves followed the protocols outlined by Xu et al. (2023), while the preparation of samples for transmission electron microscopy (TEM) followed the methods described by Xie et al. (2020). TEM images of the palisade tissue cells were taken to quantify mesophyll cell wall thickness. Samples from each treatment were measured six times to ensure accuracy.

### 2.6 Extraction and analysis of total RNA using qRT-PCR

RNA from the roots was isolated using an RNAprep Pure Plant Kit (Tiangen, Beijing, China). The qRT-PCR reaction was performed in a 20 μL volume comprising 1 μL of cDNA, 2 μL of primers, 10 μL of Green qPCR SuperMix, and 7 μL of ddH2O. The expression levels of the genes were quantified using the 2^–ΔΔCT^ method. All qRT-PCR experiments included six biological replicates. The primers employed for qRT-PCR are listed in **Supplementary Table S3**.

### 2.7 Determination of N metabolism enzyme activity and N metabolism intermediates

The NO_3_^−^ content was assayed through the nitration of salicylic acid as described by Cataldo et al. (1975). The NH_4_^+^ content was determined using the method outlined by Brautigam et al. (2007). The contents of free amino acids and soluble proteins were measured following the procedure described by Ruiz and Romero (2002).

The activities of nitrate reductase (NR), glutamine synthetase (GS), and Fd-glutamate synthase (Fd-GOGAT) were measured using the methodology of Hu et al. (2016). In addition, the NiR activity was determined following a previously established method (Seith et al., 1994).

### 2.8 MDA, H_2_O_2_, and O_2_^−^ contents

The concentration of MDA in the roots was measured following the procedures described by Gou et al. (2020), while H_2_O_2_ and O_2_^−^ content were determined using the methods described by Zhang et al. (2008).

### 2.9 Hormone contents in the roots

A sample of freeze-dried root (1.0 g) was purified and analyzed using high-performance liquid chromatography to quantify the amounts of IAA, GA_3_, and ABA, following the methods outlined by Almeida Trapp et al. (2014).

### 2.10 Data analysis

Statistical evaluation was conducted using one-way ANOVA followed by Duncan’s *post hoc* test using the SPSS software (version 17.0, IBM, USA). Regression analysis and curve fitting were performed using OriginPro (2021, OriginLab Corporation, USA). Significant differences were considered at a P-value ≤ 0.05.

## 3 Results

### 3.1 Plant growth and root morphology

**Figure 1:**
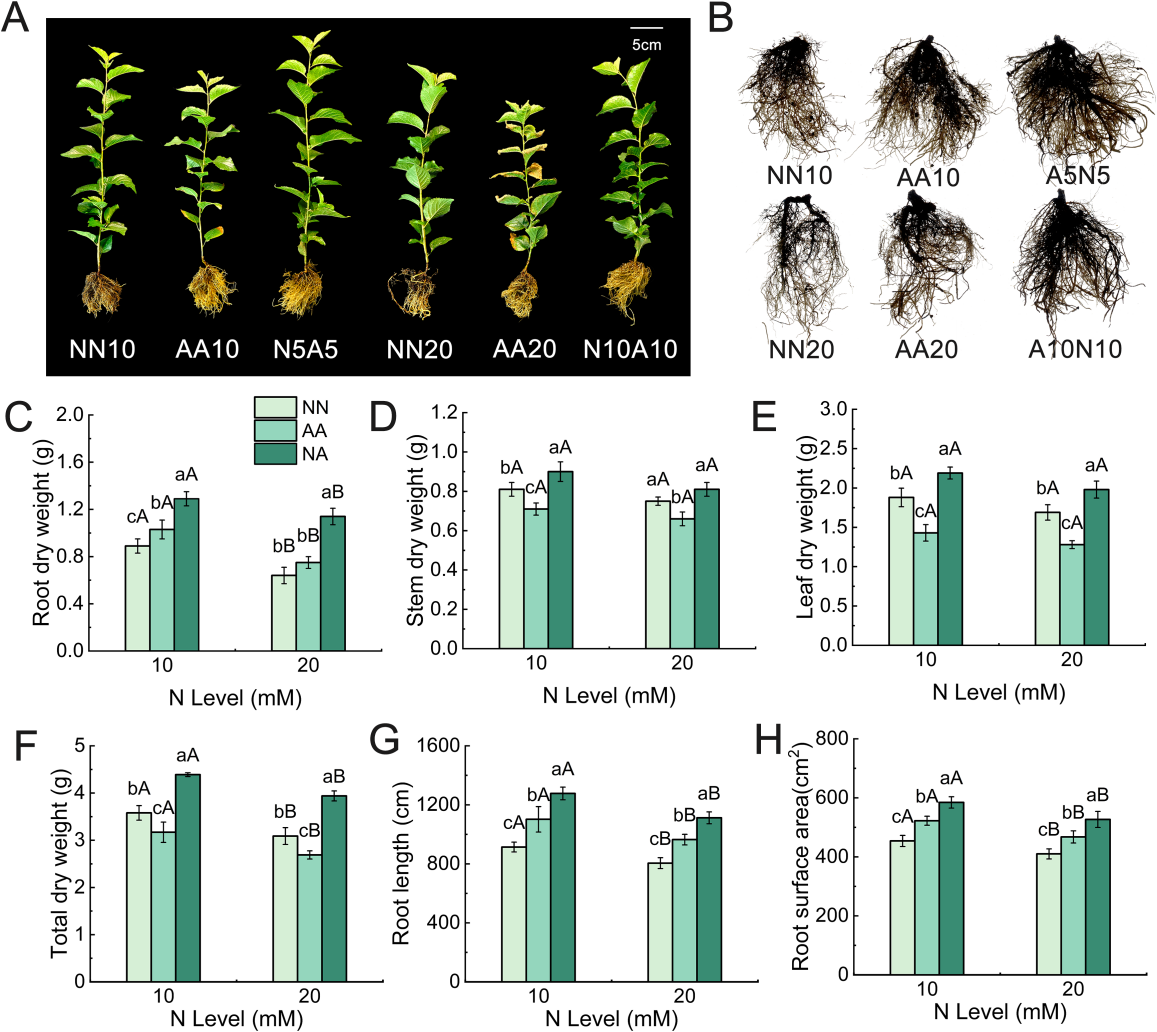
The growth and root morphology of Gisela 6 rootstock under different N treatments. Growth phenotypes of rootstock **(A)**, root morphology **(B)**, root dry weight **(C)**, stem dry weight **(D)**, leaf dry weight **(E)**, total dry weight **(F)**, root length **(G)**, and root surface area **(H)**. Different lowercase (capital) letters indicate significant differences between N forms (N levels) under the same N levels (N forms) (P < 0.05). The data are presented as means ± standard deviation (n = 5). NN10, medium N with nitrate; AA10, medium N with ammonium; N5A5, medium N with mixed N; NN20, high N with nitrate; AA20, high N with ammonium; N10A10, high N with mixed N. NN, nitrate treatment; AA, ammonium treatment; NA, mixed N treatment.

Both the N levels and N forms significantly affected the growth and root morphological development of cherry rootstock (**Figures 1A, B**). Compared with the medium N (MN) treatment, the high N (HN) treatment resulted in significant decreases in the root biomass, total dry weight, root length, and root surface area of cherry rootstock (**Figures 1D–F**). Specifically, root biomass decreased by 27.99%, 27.42%, and 11.63% under nitrate N, ammonium N, and mixed N treatments, respectively (**Figure 1C**). Among the different N forms, cherry rootstocks under the mixed N treatment exhibited significantly higher biomass of various organs, root length, and root surface area compared with those under the nitrate N and ammonium N treatments (**Figures 1D–H**). Analysis of endogenous root hormones revealed that the IAA content was lowest under the nitrate N treatment, while the ABA content was highest (**Supplementary Table S4**). Conversely, the mixed N treatment resulted in the highest IAA and GA_3_ contents and the lowest ABA content. The HN treatment reduced the IAA and GA_3_ contents in the roots while increasing the ABA content.

### 3.2 N content and accumulation

**Figure 2:**
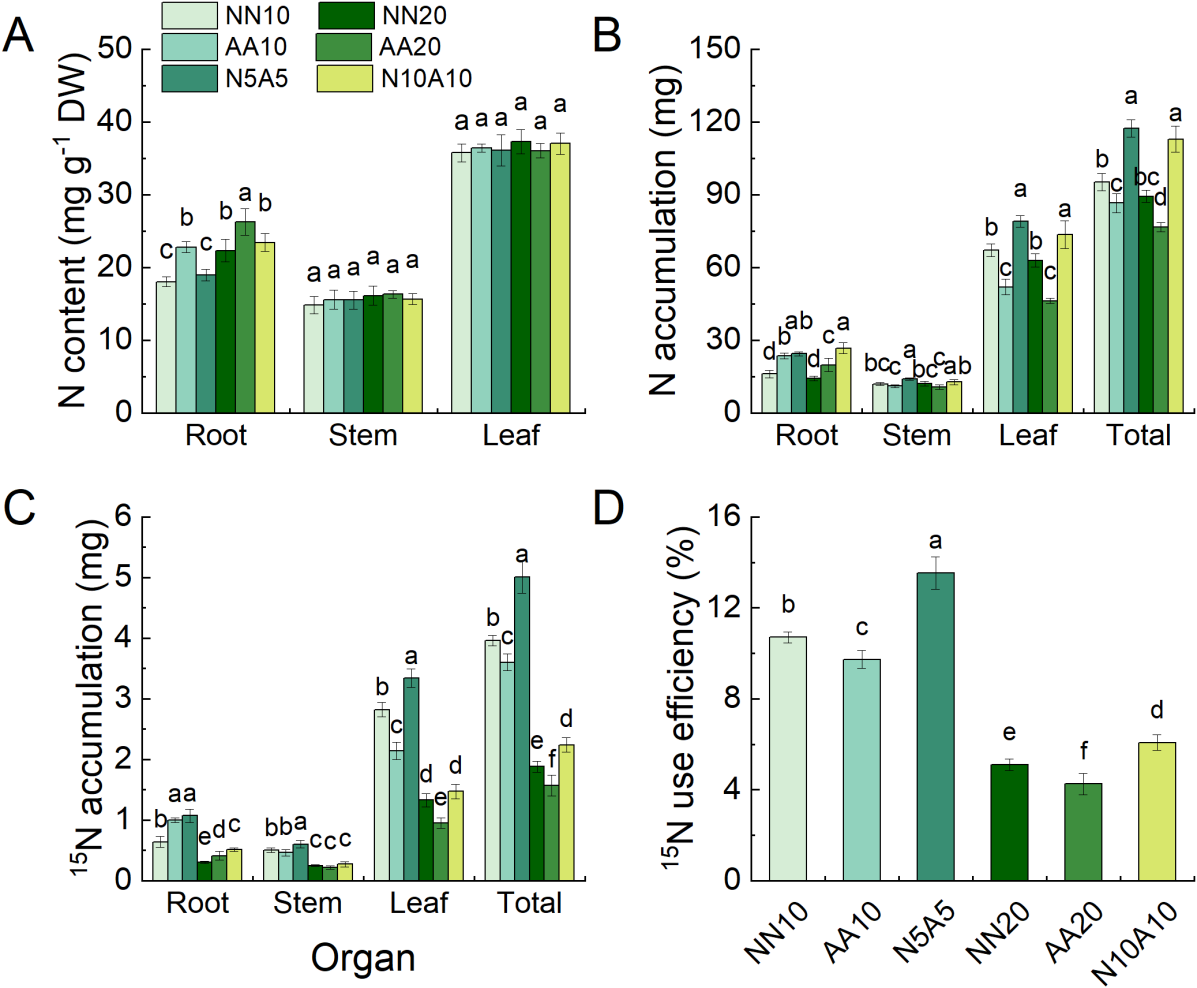
The N content **(A)**, N accumulation **(B)**, ^15^N accumulation **(C)**, and ^15^N use efficiency **(D)** of Gisela 6 rootstock under different N treatments. Different letters indicate significant differences between different N treatments (P < 0.05). The data are presented as means ± standard deviation (n = 5). NN10, medium N with nitrate; AA10, medium N with ammonium; N5A5, medium N with mixed N; NN20, high N with nitrate; AA20, high N with ammonium; N10A10, high N with mixed N.

Under the HN treatment, the N content in the roots of cherry rootstock was significantly higher than that under the MN treatment (**Figure 2**). However, there were no significant differences in N content between the stems and leaves among the various treatments (**Figure 2A**). Nevertheless, compared with the MN treatment, N accumulation in all organs and the whole plant under high N treatment was not significantly increased (**Figure 2B**). The total N accumulation in cherry rootstock was highest under the mixed N treatment. The whole-plant ^15^N accumulation and ^15^N utilization efficiency were lowest under the ammonium N treatment (**Figure 2C**), while the ^15^N utilization efficiency was highest under the mixed N treatment (**Figure 2D**).

### 3.3 N uptake

**Figure 3:**
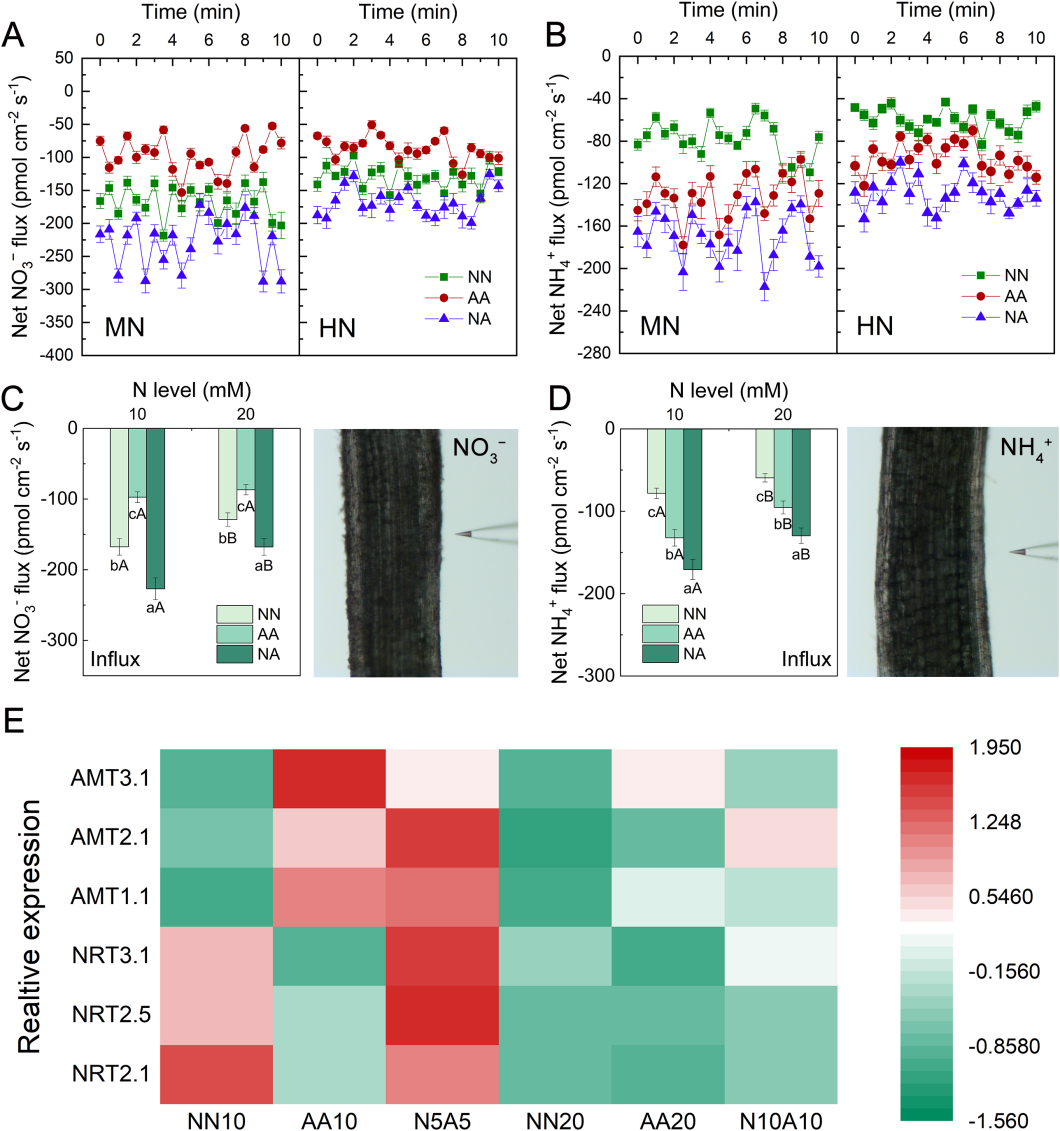
The NO_3_^−^ and NH_4_^+^ inflow rates and the relative expression levels of N uptake genes of the roots of Gisela 6 rootstock under different N treatments. The influx of NO_3_^−^ and NH_4_^+^**(A, B)**, average influx rates of NO_3_^−^ and NH_4_^+^**(C, D)**, and the relative expression levels of N uptake genes of rootstock roots **(E)**. Different lowercase (capital) letters indicate significant differences between N forms (N levels) under the same N levels (N forms) (P < 0.05). The data are presented as means ± standard deviation (n = 6). NN, nitrate treatment; AA, ammonium treatment; NA, mixed N treatment.

Among the different N levels, the mixed N treatment resulted in the highest net influx rates of NO_3_^−^ and NH_4_^+^ on the root surface as well as the highest average flux rates of NO_3_^−^ and NH_4_^+^ within 10 minutes (**Figures 3A, B**). In addition, the HN treatment reduced the net influx rates of NO_3_^−^ and NH_4_^+^ in the roots (**Figures 3C, D**). We examined the expression levels of nitrate and ammonium transporters in the seedling roots. The results indicated that the HN treatment significantly decreased the expression levels of both the nitrate and ammonium transporters (**Figure 3E**). The nitrate N treatment reduced ammonium transporter expression, while the ammonium N reduced nitrate transporter expression. In contrast, the mixed N treatment maintained high expression of both nitrate and ammonium transporters in the roots (**Figure 3E**), consistent with the results of ion flux rates on the root surface.

### 3.4 N assimilation

**Figure 4:**
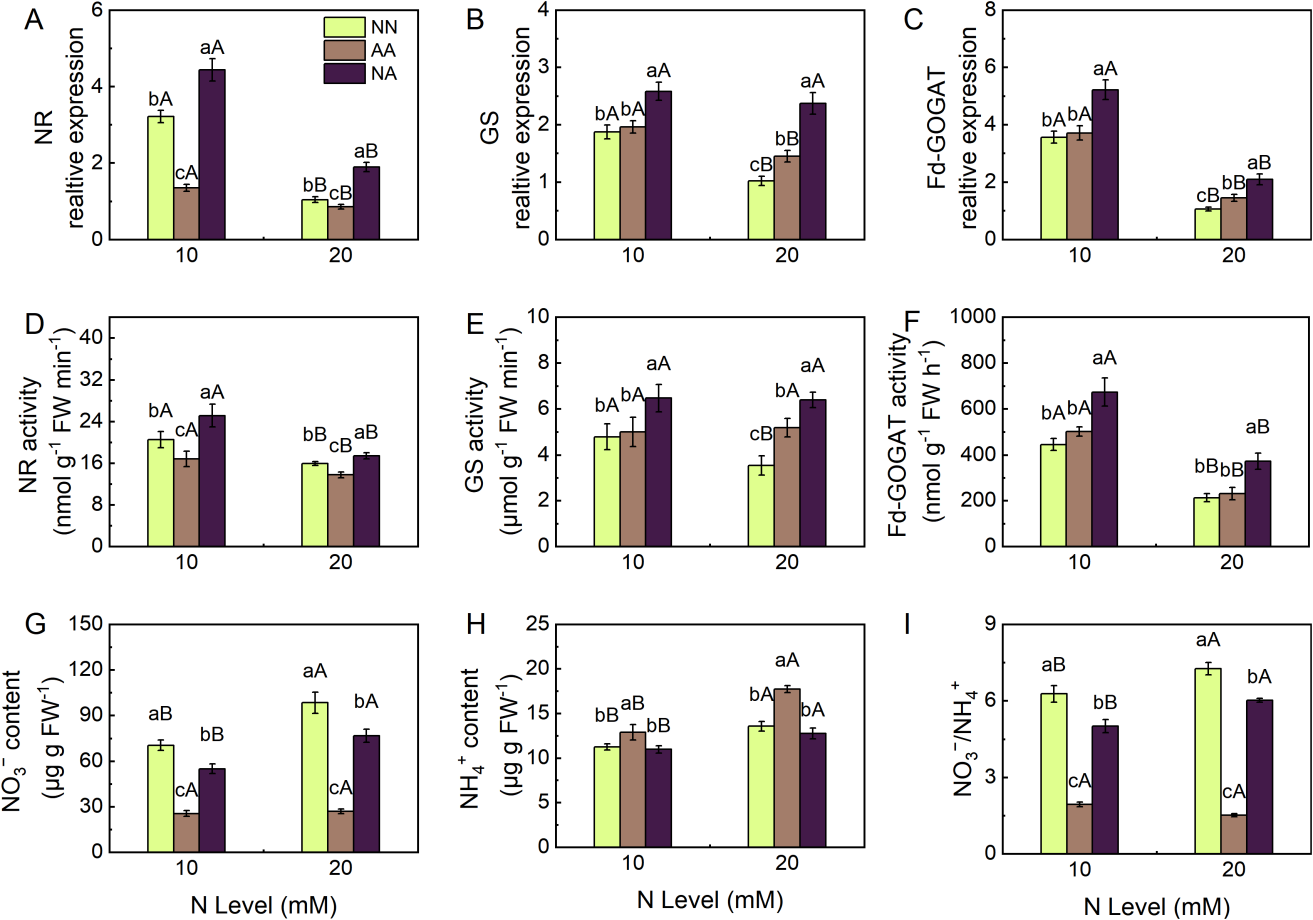
The effects of different N treatments on the expression of N metabolism genes, activities of key enzymes, and the content of N metabolism intermediates in the leaves of Gisela 6 rootstock. NR relative expression **(A)**, GS relative expression **(B)**, Fd-GOGAT relative expression **(C)**, NR activity **(D)**, GS activity **(E)**, Fd-GOGAT **(F)**, NO_3_^−^ content **(G)**, NH_4_^+^ content **(H)**, and NO_3_^−^/NH_4_^+^**(I)**. Different lowercase (capital) letters indicate significant differences between N forms (N levels) under the same N levels (N forms) (P < 0.05). The data are presented as means ± standard deviation (n=5). NN, nitrate treatment; AA, ammonium treatment; NA, mixed N treatment.

Compared with the MN treatment, the HN treatment significantly suppressed the transcription of nitrate reductase (NR) activity, and glutamate synthase (Fd-GOGAT) genes, leading to corresponding decreases in the activities of NR and Fd-GOGAT enzymes in the leaves of cherry rootstock (**Figures 4A, C**). The HN treatment increased the contents of NH_4_^+^ and free amino acids in the leaves while reducing the content of soluble proteins (**Figures 4D, E**). Except for the ammonium N treatment, both the nitrate N and mixed N treatments led to an increase in the NO_3_^−^/NH_4_^+^ in the leaves under HN conditions (**Figure 4F**). Among the different N forms, the nitrate N treatment resulted in the highest NO_3_^−^ content and NO_3_^−^/NH_4_^+^ in the leaves of cherry rootstock (**Figures 4G, I**), while the ammonium N treatment resulted in the highest NH_4_^+^ content and the lowest NO_3_^−^/NH_4_^+^ ratio (**Figure 4H**). The coordinated upregulation of NR, GS, and GOGAT expression under the mixed N treatment directly resulted in the highest enzymatic activities of NR, GS, and Fd-GOGAT, as well as the highest accumulation of free amino acids and soluble proteins in the leaves (**Figures 4A–C**). Neither the N level nor the N form significantly affected the nitrite reductase (NiR) gene expression or enzyme activity in the leaves (**Supplementary Figure S1**).

### 3.5 N allocation and photosynthetic N use efficiency (PNUE)

**Figure 5:**
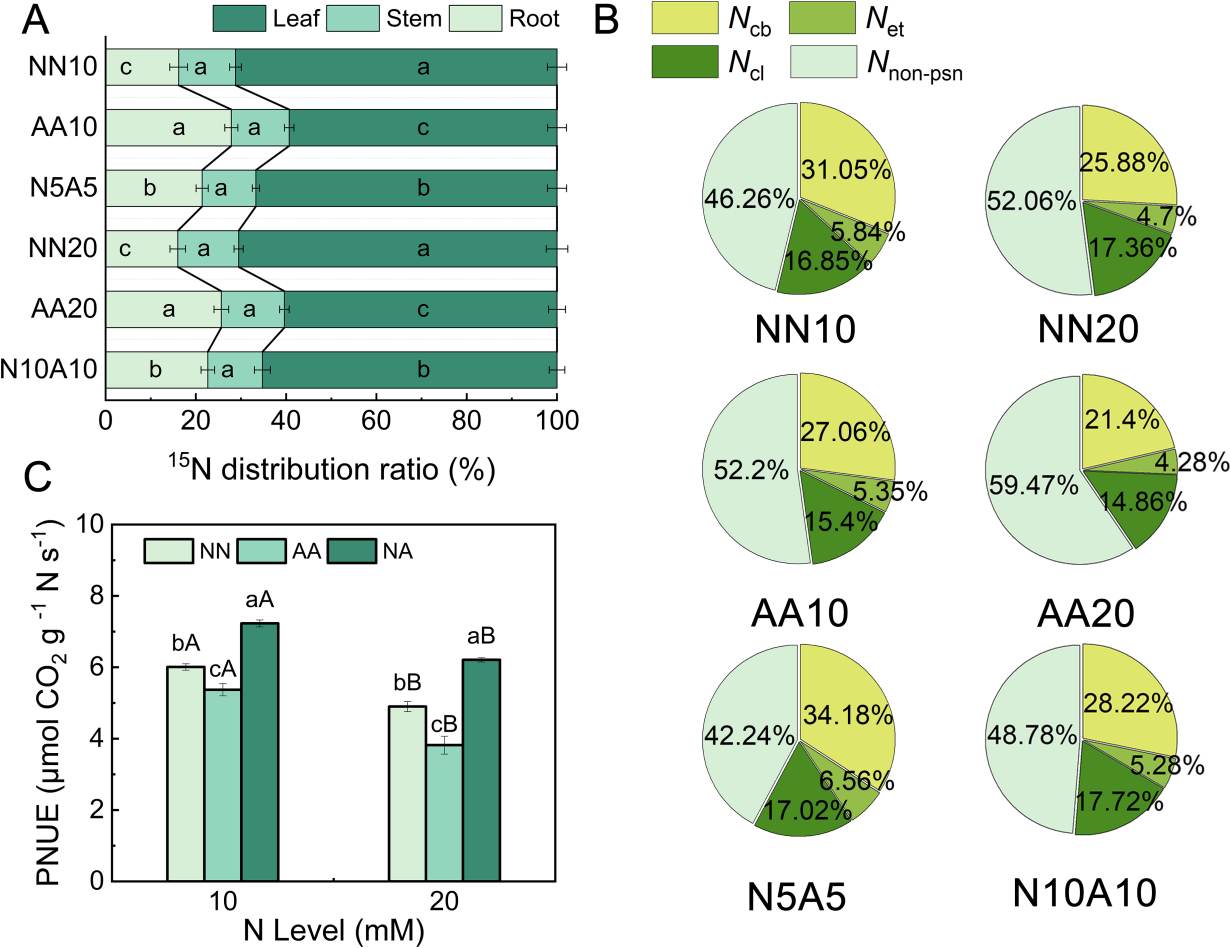
The effects of different N treatments on the ^15^N distribution ratio **(A)**, photosynthetic N allocation **(B)**, and PNUE **(C)** of Gisela 6 rootstock. Different lowercase (capital) letters indicate significant differences between N forms (N levels) under the same N levels (N forms) (P < 0.05). The data are presented as means ± standard deviation (n=5). NN10, medium N with nitrate; AA10, medium N with ammonium; N5A5, medium N with mixed N; NN20, high N with nitrate; AA20, high N with ammonium; N10A10, high N with mixed N.

We measured the N allocation in various organs of cherry rootstock using ^15^N isotopes (**Figure 5A**). The nitrate N treatment significantly decreased N allocation to the roots and increased N allocation to the leaves. By contrast, the ammonium N treatment resulted in an opposite trend, with the lowest N allocation to leaves and the highest to roots. There were no significant differences in N allocation among organs at different N levels.

We investigated the changes in the allocation proportions of photosynthetic (*N*_psn_) and non-photosynthetic N (*N*_non-psn_) in the leaves (**Figure 5B**). At the same N level, the distribution proportions of carboxylation N (*N*_cb_), electron transfer N (*N*_et_), and light capture N (*N*_lc_) as well as the PNUE were the lowest in cherry rootstock leaves under the ammonium N treatment. By contrast, the distribution proportions of *N*_psn_ and PNUE were the highest in cherry rootstock leaves under the mixed N treatment. Compared with the MN treatment, the HN treatment significantly reduced the allocation proportion of *N*_cb_ and *N*_et_ in leaves as well as PNUE (**Figure 5C**). We conducted an additional analysis to explore the connection between PNUE and N allocation in various organs and the allocation proportion of *N*_psn_ (**Supplementary Figure S2**). The results showed that PNUE was significantly positively correlated with the allocation proportions of *N*_cb_, *N*_et_, and *N*_lc_, and significantly negatively correlated with the allocation proportion of *N*_non-psn_. No statistically significant relationship was observed between PNUE and N allocation proportions in roots or leaves (**Supplementary Figures S2E, F**).

### 3.6 Leaf structure and photosynthetic limitation

**Figure 6:**
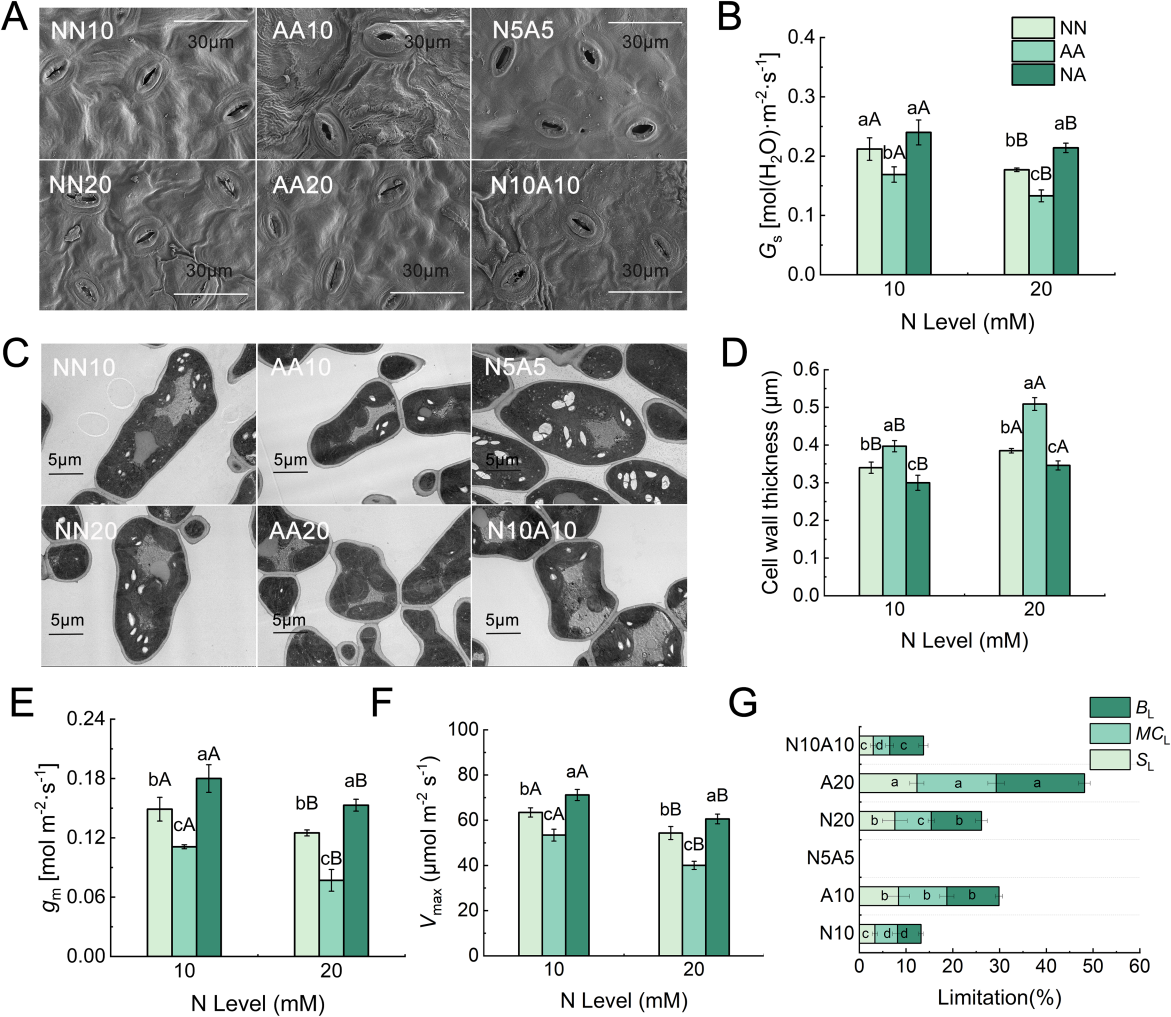
The effects of different N treatments on stomatal conductance **(A, B)**, cell wall thickness **(C, D)**, mesophyll conductance **(E)**, maximum carboxylation rate **(F)**, and photosynthetic limitation **(G)** of Gisela 6 rootstock. Different lowercase (capital) letters indicate significant differences between N forms (N levels) under the same N levels (N forms) (P < 0.05). The data are presented as means ± standard deviation (n = 5). NN10, medium N with nitrate; AA10, medium N with ammonium; N5A5, medium N with mixed N; NN20, high N with nitrate; AA20, high N with ammonium; N10A10, high N with mixed N; NN, nitrate treatment; AA, ammonium treatment; NA, mixed N treatment.

We investigated the mechanisms underlying the changes in leaf photosynthesis in the N treatments to explain the variation in PNUE. Scanning electron microscope (SEM) observations showed that the degree of stomatal opening, from highest to lowest, was in leaves in the mixed N, nitrate N, and ammonium N treatments (**Figures 6A, B**). Transmission electron microscopy showed that the ammonium N treatment increased cell wall thickness, while the mixed N treatment resulted in the lowest cell wall thickness and the highest mesophyll conductance (**Figures 6C, D**). The analysis of photosynthetic limitations also indicated that stomatal conductance limitation (*S*_L_), mesophyll conductance limitation (*M*_CL_), and physiological and biochemical limitation (*B*_L_) were highest under the ammonium N treatment and lowest under the mixed N treatment (**Figure 6G**). The HN treatments reduced the maximum carboxylation rate (*V*_max_), stomatal conductance (*g*_s_) and mesophyll conductance (*g*_m_) while increasing *B*_L_, *S*_L_, and *M*_CL_ (**Figures 6E, F**). The highest photosynthetic limitation and the lowest *P*_n_ were observed under the AA20 treatment (**Supplementary Figure S3**).

### 3.7 Correlation analysis

**Figure 7:**
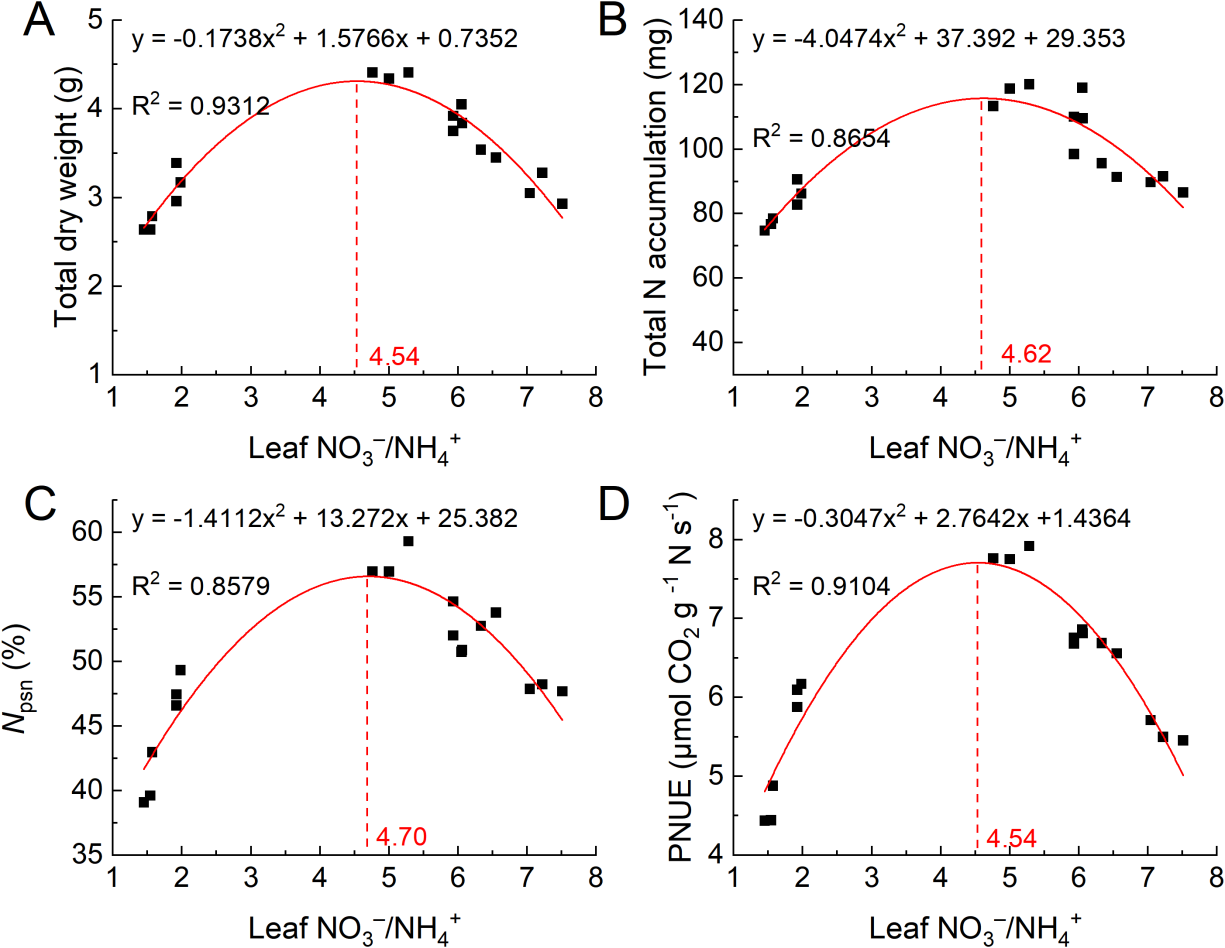
The relationship between the total dry biomass **(A)**, total N accumulation **(B)**, *N*_psn_/Leaf N **(C)**, PNUE **(D)**, *N*_psn_/Leaf N, and N/K of Gisela 6 cherry rootstock. The relationships were fitted with a quadratic polynomial regression model using OriginPro (2021, OriginLab Corporation, USA.).

We analyzed the relationships between the NO_3_^−^/NH_4_^+^ in leaves and the total dry biomass, total N accumulation, *N*_psn_/Leaf N, and PNUE of cherry rootstock (**Figure 7**). The results showed that as the NO_3_^−^/NH_4_^+^ ratio increased, the total dry biomass, total N accumulation, PNUE, and *N*_psn_/Leaf N of the seedlings initially increased, after which they decreased. Based on the fitted equations, the maximum values for total dry biomass, total N accumulation, *N*_psn_/Leaf N, and PNUE were achieved when the NO_3_^−^/NH_4_^+^ ratios were approximately 4.54, 4.62, 4.70, and 4.54, respectively. In addition, we analyzed the relationships between the NO_3_^−^/NH_4_^+^ ratio in leaves and the contents of malondialdehyde (MDA), hydrogen peroxide (H_2_O_2_), and superoxide anion radical (O_2_^−^) (**Supplementary Figure S4**). The results indicated that as the NO_3_^−^/NH_4_^+^ ratio increased, the contents of MDA, H_2_O_2_, and O_2_^−^ in the leaves followed a trend of first decreasing and then increasing, reaching their lowest points at NO_3_^−^/NH_4_^+^ ratios of 4.76, 4.57, and 4.86, respectively. These results suggest that a NO_3_^−^/NH_4_^+^ ratio of 4–5 in leaves is optimal.

We also analyzed the relationship between leaf N allocation and the leaf antioxidant system (**Supplementary Figure S5**). The results indicated that the allocation proportion of *N*_psn_ gradually decreased with increasing MDA content, H_2_O_2_ content, and superoxide O_2_^−^ content in leaves, showing a significant negative correlation. By contrast, the allocation proportion of *N*_non-psn_ was significantly positively correlated with the MDA content, H_2_O_2_ content, and O_2_^−^ content.

## 4 Discussion

The root system is responsible for absorbing water and nutrients, and its architecture and absorptive capacity directly influence the growth status of plants. Most previous studies have found that NH_4_^+^ being the sole or primary N source often has detrimental effects on root growth (Britto and Kronzucker, 2002; Esteban et al., 2016). However, our results demonstrated that compared with nitrate N treatment, the ammonium N treatment significantly increased the root dry weight, root length, and surface area of the cherry rootstock, suggesting that different plants may have species-specific preferences for N forms. As a core regulator of root development, IAA promotes root growth through multiple mechanisms such as enhancing meristematic activity in root tips, driving lateral root initiation, and by regulating cell wall acidification and loosening (Saini et al., 2013). Conversely, abscisic acid (ABA) may inhibit root growth by suppressing the cell cycle in root tips and antagonizing IAA-mediated cell wall acidification (Chen et al., 2020). Consequently, the optimal root growth observed under mixed N supply is likely associated with the highest root IAA content and the lowest ABA content. The inhibitory effect of HN on root growth may also be linked to reduced IAA and increased ABA levels. This is consistent with the findings of Chen et al. (2018) in pear trees, indicating that root growth responds negatively to excessive N.

Beyond root growth, the form and level of N supply profoundly impacted N acquisition and utilization. Compared with treatments with a single N form, the total N accumulation and ^15^N utilization were highest under the combined treatment of nitrate and ammonium N. The results for NMT and the gene expression levels related to N uptake suggest that the supply of a single N form has a negative impact on the uptake of other forms. However, the combined treatment of nitrate and ammonium N increased the expression levels of nitrate transporter proteins and ammonium transporter proteins in the roots, increasing the net influx rates of NO_3_^−^ and NH_4_^+^ at the root surface and thereby enhancing N uptake in cherry rootstock, which was similar to that of Li et al. (2023) in oilseed rape. In addition to enhancing N uptake, the mixed N treatment also significantly improved N assimilation. This was evidenced by an increase in the transcript levels and activities of NR, GS and Fd-GOGAT in leaves, along with elevated soluble protein content (**Figure 4**). These findings align with previous studies demonstrating that combinations of different N sources can reduce the negative feedback regulation of single N forms on NR and GS, thereby promoting N assimilation (Rivero-Marcos et al., 2025). Notably, the HN treatment exhibited a significant inhibitory effect on both N uptake and assimilation, consistent with our previous observations in apples (Xu et al., 2024), and likely contributing to the stagnation in N accumulation despite increased external N supply.

The levels and forms of N supply also influence the distribution of N within cherry rootstock plants and their leaves, as well as the PNUE. Our results indicate that the proportion of photosynthetic N allocation in leaves was closely associated with the contents of malondialdehyde (MDA) and reactive oxygen species (ROS), suggesting that oxidative damage was a key regulator of leaf N allocation. A similar trade-off in leaf N allocation between photosynthesis and stress defense was also observed in rice under drought stress (Zhong et al., 2019). This aligns with previous research indicating that plants can enhance leaf toughness and chemical defense capabilities to resist stress by regulating the allocation of N to the leaves (Feng et al., 2009; Sharwood et al., 2017). Moreover, we found that the contents of MDA and ROS in leaves were closely associated with the NO_3_^−^/NH_4_^+^. An imbalance in the NO_3_^−^/NH_4_^+^ ratio triggered an accumulation of MDA, H_2_O_2_, and O_2_^−^, thereby reducing the proportion of *N*_psn_, and ultimately leading to significant reductions in net photosynthetic rate and PNUE in the leaves. These findings are consistent with the results reported by Chen et al. (2023) in citrus. Under the mixed N treatment, the NO_3_^−^/NH_4_^+^ ratio in the leaves remained within an optimal range, with the lowest MDA and ROS contents in the leaves. Consequently, the leaves allocated more N to photosynthetic parts, further optimizing photosynthesis and enhancing PNUE. Li et al. (2023) made similar discoveries in oilseed rape, finding that nitrate could alleviate ammonium toxicity by coordinating rhizosphere and cellular pH. These results suggest that maintaining an appropriate leaf NO_3_^−^/NH_4_^+^ ratio is crucial for optimizing leaf N allocation and improving PNUE.

We also analyzed the changes in photosynthetic limitation to determine the physiological mechanisms by which various N treatments regulated PNUE. Observations from leaf scanning electron microscopy and gas exchange measurements revealed that the ammonium N treatment led to the greatest degree of stomatal closure and the highest stomatal limitation (*S*_L_), while the mixed N treatment resulted in the greatest degree of stomatal opening, the highest value of *g*_s_, and the lowest value of *S*_L_. Stomatal closure under ammonium stress is likely a response to the accumulation of reactive oxygen species in the leaves, as plants close their stomata to resist stress under adverse conditions (Ouyang et al., 2017). *G*_m_ is closely related to mesophyll anatomical characteristics, with cell wall resistance accounting for approximately half of the mesophyll resistance (Evans et al., 2009). Accordingly, the highest *g*_m_ under the mixed N treatment correlated with the lowest mesophyll cell wall thickness. Since leaf cell wall thickness was correlated with non-photosynthetic N (Xie et al., 2020), the increased allocation to non-photosynthetic N under high N and ammonium supply led to thicker mesophyll cell walls, which was a structural adaptation to stress. This alteration restricted the conduction of CO_2_ within the leaf and enhanced *M*_CL_. This finding is consistent with the results reported by Shang et al. (2019) in ozone-stressed poplar, indicating that plants allocate more N to cell wall structures as an adaptation to stress conditions. Under the mixed N treatment, the *g*_s_, *g*_m_, and *V*_max_ values of cherry rootstock leaves were maximized, resulting in the lowest photosynthetic limitation and the highest PNUE.

In summary, our results indicate that a high N supply inhibited the growth of roots, impeding N uptake and assimilation and leading to an imbalance in the NO_3_^−^/NH_4_^+^ ratio and oxidative damage to the leaves, and increased photosynthetic limitation, thereby adversely affecting PNUE and N absorption and utilization. Compared with the treatments using a single N source, the combined application of nitrate N and ammonium N promoted root growth and maintained higher transcription levels of N uptake genes (*NRT2.5*, *NRT3.1*, *AMT1.1*, and *AMT2.1*) in cherry rootstock roots and higher N metabolism enzyme activities (NR, GS, and Fd-GOGAT), thereby facilitating N uptake and assimilation and enhancing N utilization efficiency. The mixed N treatment balanced the NO_3_^−^/NH_4_^+^ ratio in leaves, reduced oxidative damage, and resulted in the allocation of more N to photosynthetic N components in leaves; this increased *g*_m_ and *V*_max_, reduced photosynthetic limitation, and thus improved PNUE. In conclusion, appropriate N supply levels and the combined application of nitrate and ammonium N can enhance N uptake and assimilation, optimize N allocation, reduce leaf oxidative damage and photosynthetic limitations, improve leaf photosynthetic capacity and PNUE, and ultimately promote the growth of cherry rootstock.

## Data Availability

The original contributions presented in the study are included in the article/**Supplementary Material**. Further inquiries can be directed to the corresponding authors.
