# Supplementary material for: Mixed nitrogen forms enhance growth and photosynthetic nitrogen use efficiency by optimizing nitrogen metabolism and leaf N allocation in Gisela 6 cherry rootstock
Source: Front Plant Sci. 2025 Nov 25;16:1696713. doi: 10.3389/fpls.2025.1696713 (PMC12685864; doi:10.3389/fpls.2025.1696713)
Supplement: Supplementary file 1 [file Supplementaryfile1.docx]

**Table S1** The effects of different N treatments on total dry weight and plant height of Gisela 6 rootstocks.

| Treatment | Total dry weight (g) | plant height (cm) |
| --- | --- | --- |
| NN5 | 2.53±0.08cA | 22.73±0.71cA |
| NN10 | 3.24±0.14aA | 29.40±0.92aA |
| NN15 | 3.06±0.11abA | 27.63±0.85bA |
| NN20 | 2.87±0.18bA | 26.39±0.88bA |
| AA5 | 2.30±0.07bB | 19.73±1.31bB |
| AA10 | 2.86±0.08aB | 25.55±0.70aB |
| AA15 | 2.36±0.15bB | 19.60±1.30bB |
| AA20 | 2.04±0.12cB | 16.52±1.04cB |

Note: Each treatment had five biological replicates and values are expressed as means ± standard deviations (SD, n = 5). Different lowercase (capital) letters indicate significant differences between N forms (N levels) under the same N levels (N forms) (P < 0.05).

**Table S2** Summary of nitrogen treatments applied in the experiment.

| Treatment | Nitrogen sources | Total N Concentration (mM) | Abbreviations |
| --- | --- | --- | --- |
| Medium N + Nitrate | Ca(NO_3_)_2_ | 10 | NN10 |
| Medium N + Ammonium | (NH_4_)_2_SO_4_ | 10 | AA10 |
| Medium N + Mixed N | Ca(NO_3_)_2_ + (NH_4_)_2_SO_4_  (1:1 NH_4_^+^:NO_3_⁻) | 10 | N5A5 |
| High N + Nitrate | Ca(NO_3_)_2_ | 20 | NN20 |
| High N + Ammonium | (NH_4_)_2_SO_4_ | 20 | AA20 |
| High N + Mixed N | Ca(NO_3_)_2_ + (NH_4_)_2_SO_4_  (1:1 NH_4_^+^:NO_3_⁻) | 20 | N10A10 |

**Table S3** The compositions of measuring solution under different nitrogen treatments.

| Treatment | Ca(NO_3_)_2_ (mM) | (NH_4_)_2_SO_4_ (mM) | PH |
| --- | --- | --- | --- |
| NN10 | 5 | 0.05 | 6.0 |
| AA10 | 0.05 | 5 | 6.0 |
| N5A5 | 2.5 | 2.5 | 6.0 |
| NN20 | 10 | 0.05 | 6.0 |
| AA20 | 0.05 | 10 | 6.0 |
| N10A10 | 5 | 5 | 6.0 |

**Table S4** The effects of different N treatments on endogenous hormone content in roots of Gisela 6 rootstocks.

|  | Treatment | IAA content（ng/g.FW） | GA_3_ content（ng/g.FW） | ABA content（ng/g.FW） |
| --- | --- | --- | --- | --- |
| N10 | NN | 13.67±0.91bA | 1.67±0.08bA | 33.11±1.96aB |
|  | AA | 20.52±1.20aA | 1.61±0.07bA | 22.71±0.93bB |
|  | NA | 22.34±0.77aA | 2.02±0.14aA | 18.23±0.78cB |
| N20 | NN | 10.31±0.81cB | 1.22±0.04cB | 40.83±1.45aA |
|  | AA | 16.32±0.97bB | 1.53±0.05bA | 28.87±0.99bA |
|  | NA | 20.37±1.25aA | 1.74±0.10aB | 22.37±1.22cA |

Note: Each treatment had five biological replicates and values are expressed as means ± standard deviations (SD, n = 5). Different lowercase (capital) letters indicate significant differences between N forms (N levels) under the same N levels (N forms) (P < 0.05).

**Table S5** | Primer sequences for qRT-PCR.

| Gene  name | Forward sequence of the primers  (5′→3′) | Reverse sequence of the primers  (5′→3′) |
| --- | --- | --- |
| NRT2.1 | AGCTTCACACAGCTGGAATC | CAAAGTACCTGGCTGCTCTATC |
| NRT2.5 | GCATCCATTGCTGTGATGATTT | CCTCCTCCTGTCATACCAGATA |
| NRT3.1 | CCACGCGTCCCTTGATATT | GGACGACTTTGCCTTTCTCT |
| AMT1.1 | TGGCCGAGAAGGTGAAATAC | AACAGCGCCGTGAAGATAA |
| AMT2.1 | GGAACATAGTCTCCACCACAAT | TGAACAGCATCGTCTCCAATAA |
| AMT3.1 | GTGCATGGAGAAGAGGCATA | TGTGACGTATCATCGGAGTAAAG |
| NR | CCCAGCCTGAGAAACTCATT | ATCTCTCCTTTGTGTCGCTTAC |
| GS | CCATTTGACTCAACACCCTACT | AGGAATGGAGAGCAGGAATAAC |
| NiR | GGAATCGACCCTGATGAGATTG | GGAATCGACCCTGATGAGATTG |
| Fd-GOGAT | TTGGGATGGACCTGCTTTAC | TTATCAGATGTCCGCCAATACC |
| Tubulin | GCGTTTGTGCATTGGTATGT | CTGCTCCAACTTCCTCATAGTC |


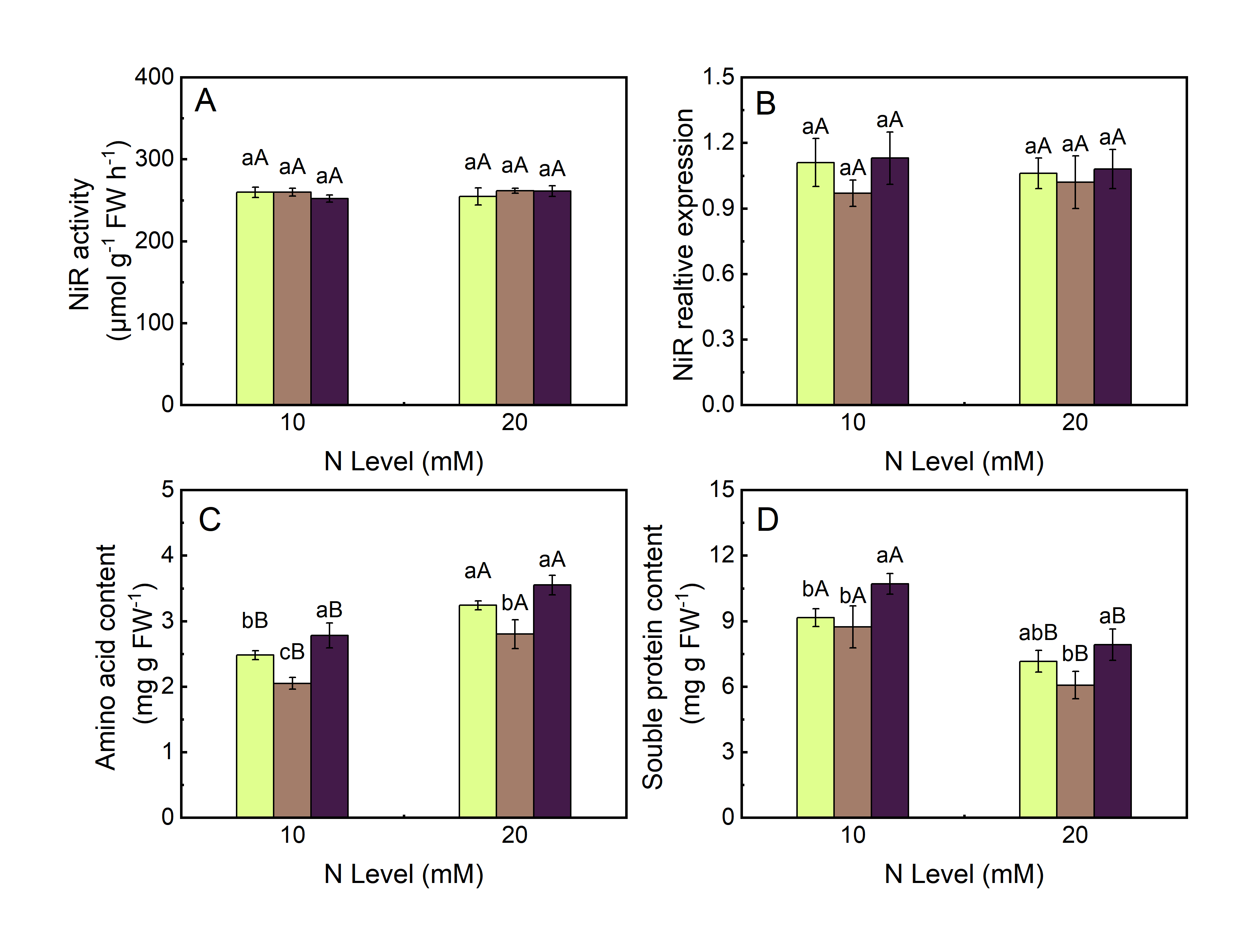


**Figure S1** The effects of different N treatments on NiR activity (A), NiR relative expression (B) , amino acid content (C), and souble protein content (D) in the leaves of Gisela 6 rootstocks. The data are presented as means ± standard deviation (n=5). Different lowercase letters indicate significant differences between N forms under the same N level, whereas different capital letters indicate significant differences between N levels under the same N form (P < 0.05) according to Duncan’s test.





**Figure S2** The relationship between N allocation in leaf (A,B,C,D), ^15^N distribution (E,F) and PNUE.





**Figure S3** The effects of different N treatments on net photosynthetic rate of Gisela 6 rootstocks.


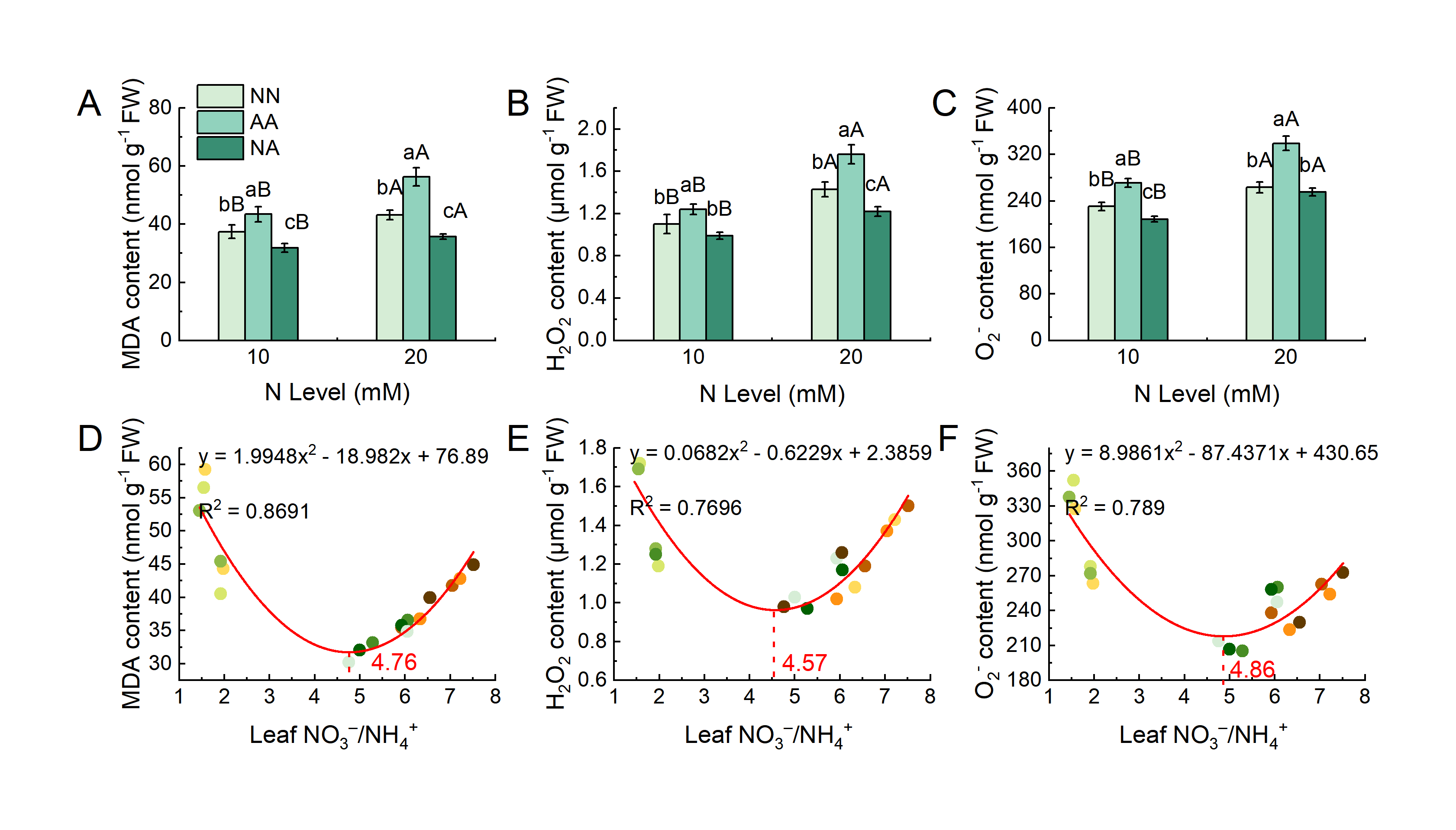


**Figure S4** The effects of different N treatments on MDA content (A), H_2_O_2_ content (B), and O_2_^−^ content in the leaves of Gisela 6 rootstocks and the relationship between MDA content (D), H_2_O_2_ content (E), O_2_^−^ content (F) and PNUE.





**Figure S5** The relationship between MDA content (A,B), H_2_O_2_ content (C,D), O_2_^−^ content (E,F) and leaf N allocation.
